# Supplementary material for: Filamentous Fungi Associated with Disease Symptoms in Non-Native Giant Sequoia (Sequoiadendron giganteum) in Germany—A Gateway for Alien Fungal Pathogens?
Source: Pathogens. 2024 Aug 23;13(9):715. doi: 10.3390/pathogens13090715 (PMC11434650; doi:10.3390/pathogens13090715)
Supplement: Supplementary file 1 [file pathogens-13-00715-s001.zip › pathogens-3089853-supplementary/Figure S1_Phylogenetic analyses.pdf]

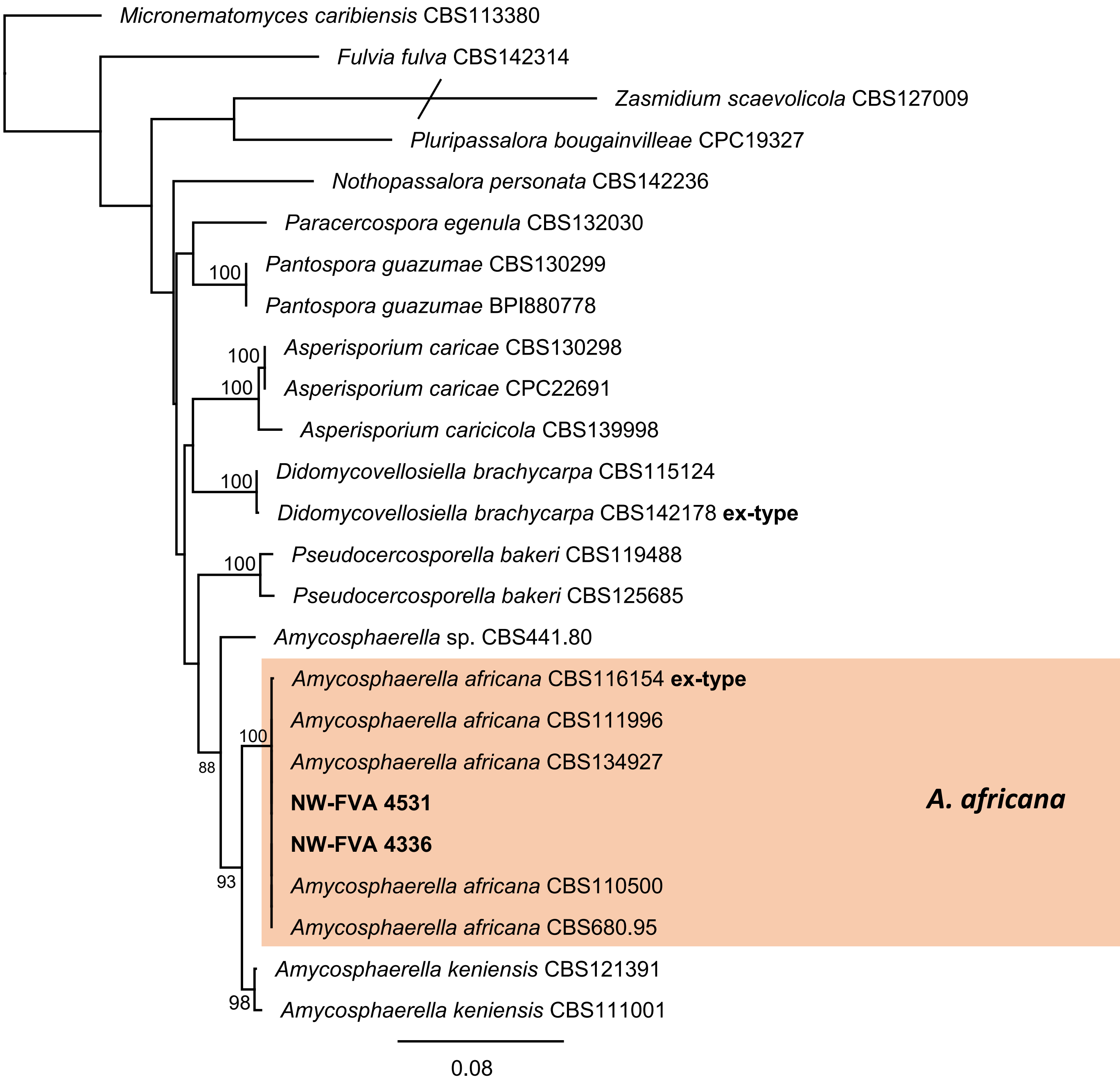


**Figure S1a.** Phylogeny obtained by Maximum likelihood analysis of the combined LSU-ITS-*RPB2* sequence alignment of species from *Mycosphaerellaceae*. ML bootstrap support values above 70 % are shown at the nodes. *Micronematomyces caribiensis* strain CBS113380 is used as outgroup. Strains analysed in this study are emphasised in bold. Branches that are crossed by diagonal lines are shortened by 50 %.

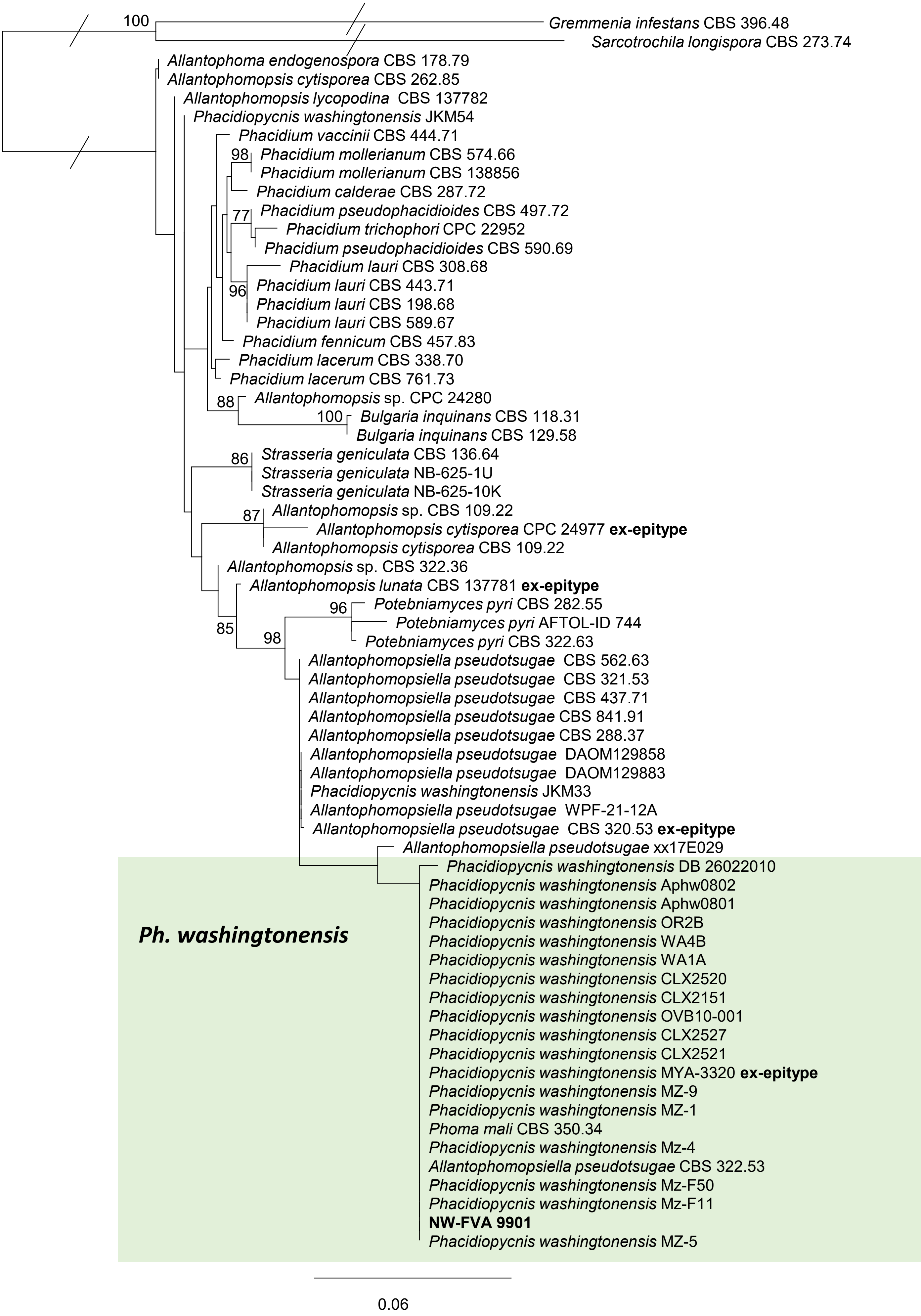

100
Gremmenia infestans CBS 396.48
Sarcotrochila longispora CBS 273.74
Allantophoma endogenospora CBS 178.79
Allantophomopsis cytisporea CBS 262.85
Allantophomopsis lycopodina CBS 137782
Phacidiopycnis washingtonensis JKM54
Phacidium vaccinii CBS 444.71
98
Phacidium mollerianum CBS 574.66
Phacidium mollerianum CBS 138856
Phacidium calderae CBS 287.72
77
Phacidium pseudophacidioides CBS 497.72
Phacidium trichophori CPC 22952
Phacidium pseudophacidioides CBS 590.69
Phacidium lauri CBS 308.68
96
Phacidium lauri CBS 443.71
Phacidium lauri CBS 198.68
Phacidium lauri CBS 589.67
Phacidium fennicum CBS 457.83
Phacidium lacerum CBS 338.70
Phacidium lacerum CBS 761.73
88
Allantophomopsis sp. CPC 24280
100
Bulgaria inquinans CBS 118.31
Bulgaria inquinans CBS 129.58
86
Strasseria geniculata CBS 136.64
Strasseria geniculata NB-625-1U
Strasseria geniculata NB-625-10K
87
Allantophomopsis sp. CBS 109.22
Allantophomopsis cytisporea CPC 24977 ex-epitype
Allantophomopsis cytisporea CBS 109.22
Allantophomopsis sp. CBS 322.36
Allantophomopsis lunata CBS 137781 ex-epitype
96
Potebniamyces pyri CBS 282.55
Potebniamyces pyri AFTOL-ID 744
85
98
Potebniamyces pyri CBS 322.63
Allantophomopsiella pseudotsugae CBS 562.63
Allantophomopsiella pseudotsugae CBS 321.53
Allantophomopsiella pseudotsugae CBS 437.71
Allantophomopsiella pseudotsugae CBS 841.91
Allantophomopsiella pseudotsugae CBS 288.37
Allantophomopsiella pseudotsugae DAOM129858
Allantophomopsiella pseudotsugae DAOM129883
Phacidiopycnis washingtonensis JKM33
Allantophomopsiella pseudotsugae WPF-21-12A
Allantophomopsiella pseudotsugae CBS 320.53 ex-epitype
Allantophomopsiella pseudotsugae xx17E029
Phacidiopycnis washingtonensis DB 26022010
Phacidiopycnis washingtonensis Aphw0802
Phacidiopycnis washingtonensis Aphw0801
Ph. washingtonensis
Phacidiopycnis washingtonensis OR2B
Phacidiopycnis washingtonensis WA4B
Phacidiopycnis washingtonensis WA1A
Phacidiopycnis washingtonensis CLX2520
Phacidiopycnis washingtonensis CLX2151
Phacidiopycnis washingtonensis OVB10-001
Phacidiopycnis washingtonensis CLX2527
Phacidiopycnis washingtonensis CLX2521
Phacidiopycnis washingtonensis MYA-3320 ex-epitype
Phacidiopycnis washingtonensis MZ-9
Phacidiopycnis washingtonensis MZ-1
Phoma mali CBS 350.34
Phacidiopycnis washingtonensis Mz-4
Allantophomopsiella pseudotsugae CBS 322.53
Phacidiopycnis washingtonensis Mz-F50
Phacidiopycnis washingtonensis Mz-F11
NW-FVA 9901
Phacidiopycnis washingtonensis MZ-5
0.06

**Figure S1b.** Phylogeny obtained by Maximum likelihood analysis of the combined LSU-ITS-*RPB2* sequence alignment of species from *Phacidiaceae*. ML bootstrap support values above 70 % are shown at the nodes. *Gremmenia infestans* strain CBS 396.48 and *Sarcotrochila longispora* strain CBS 273.74 are used as outgroup. Strains analysed in this study are emphasised in bold. Branches that are crossed by diagonal lines are shortened by 50 %.

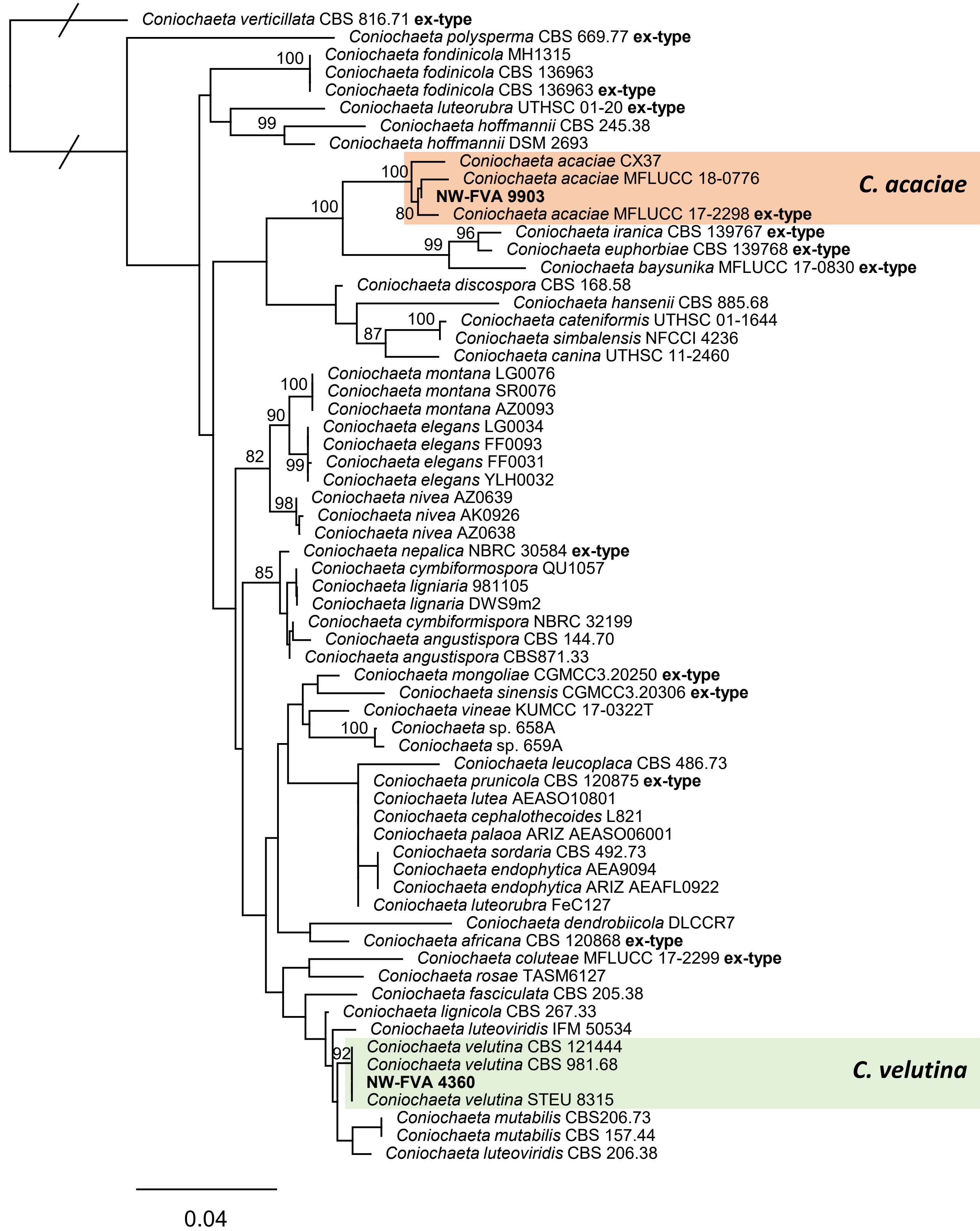


**Figure S1c.** Phylogeny obtained by Maximum likelihood analysis of the combined LSU-ITS-*EF-1α* sequence alignment of *Coniochaeta* species. ML bootstrap support values above 70 % are shown at the nodes. *Coniochaeta verticillata* strain CBS 816.71 is used as outgroup. Strains analysed in this study are emphasised in bold. Branches that are crossed by diagonal lines are shortened by 50 %.
